# Pitfall of I-131 whole body scan: a mucinous adenocarcinoma of the ovary

**DOI:** 10.11604/pamj.2020.36.72.21507

**Published:** 2020-06-08

**Authors:** Ali Sellem, Issam Msakni, Wassim Elajmi, Hatem Hammami

**Affiliations:** 1 Nuclear Medicine Department, Military Hospital of Tunis, Tunis, Tunisia,; 2 Pathology Department, Military Hospital of Tunis, Tunis, Tunisia

**Keywords:** Thyroid cancer, mucinous adenocarcinoma, ovary, radioiodine, false positive

## Abstract

False positive radioiodine uptake following thyroidectomy for differentiated thyroid cancer has been reported in some cases. A 57-year-old female patient was referred for ablative radioiodine treatment four weeks after undergoing total thyroidectomy for papillary thyroid carcinoma. Posttherapeutic I-131 scintigraphy showed uptake in the neck and large focus in the lower abdomen and pelvis. Pathology revealed a mucinous adenocarcinoma of the right ovary.

## Introduction

Radioiodine is used for treating differentiated thyroid carcinoma [1]. The presence of uptake sites on the whole body scanning (WBS) following iodine 131 (I-131) may be caused by physiological radioiodine uptake, thyroid remnants or metastasis. However, the presence of unusual lesions may cause a false-positive results on radioiodine WBS; therefore, it is imperative to carefully evaluate abnormal scans in order to appropriately manage patients with differentiated thyroid cancer (DTC) [1]. We herein report an interesting case of false positive radioiodine uptake on an ovarian mucinous adenocarcinoma.

## Patient and observation

**Figure 1:**
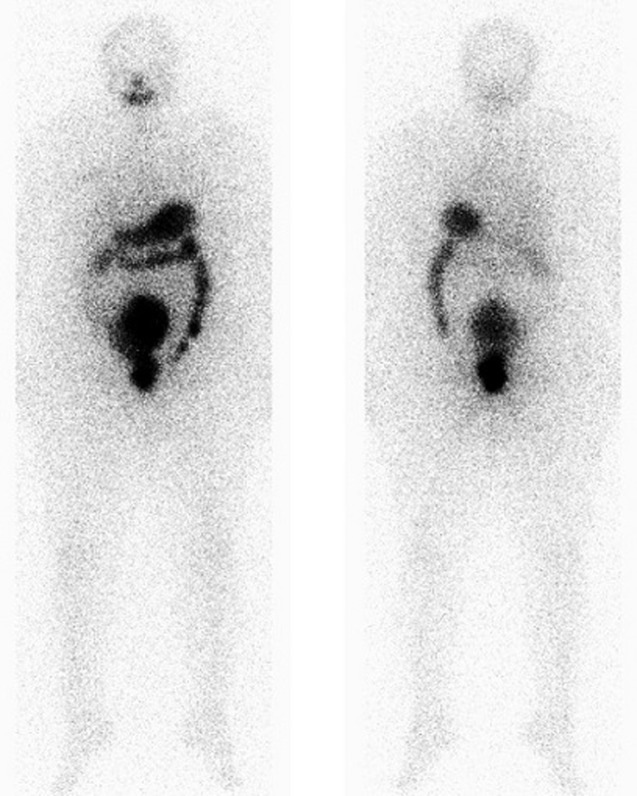
post-therapeutic whole body scanning showing a mild uptake in the neck (thyroid remnants), and a large radioiodine accumulation in the median lower abdomen and pelvis

**Figure 2:**
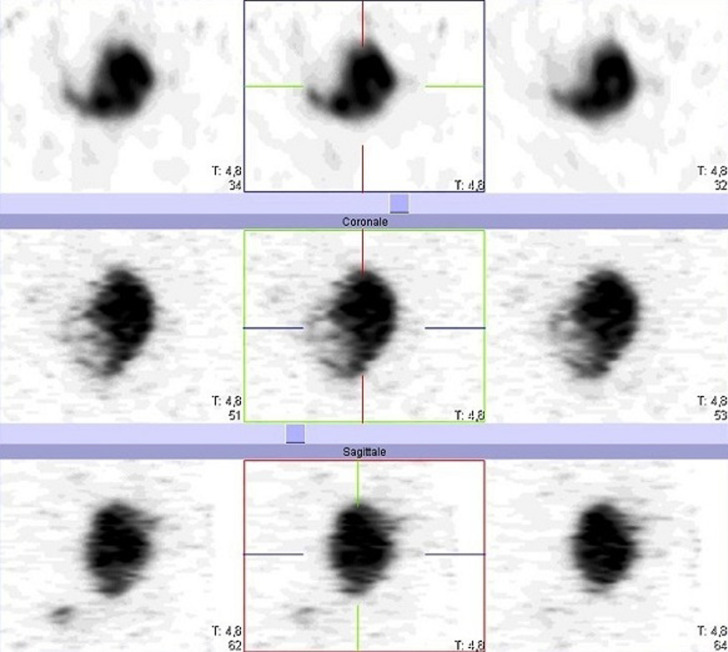
SPECT of the pelvis showing a large and heterogeneous radioiodine accumulation

**Figure 3:**
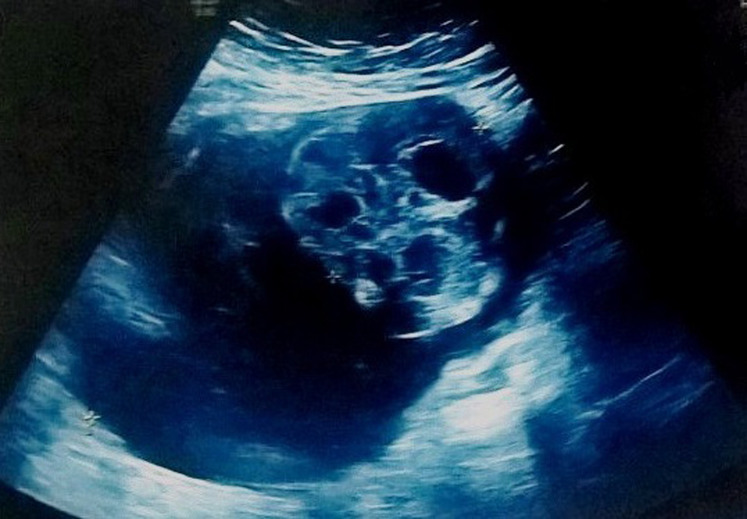
ultrasonography showing a right adnexal mass measuring 126 mm with a double tissue and cystic components

**Figure 4:**
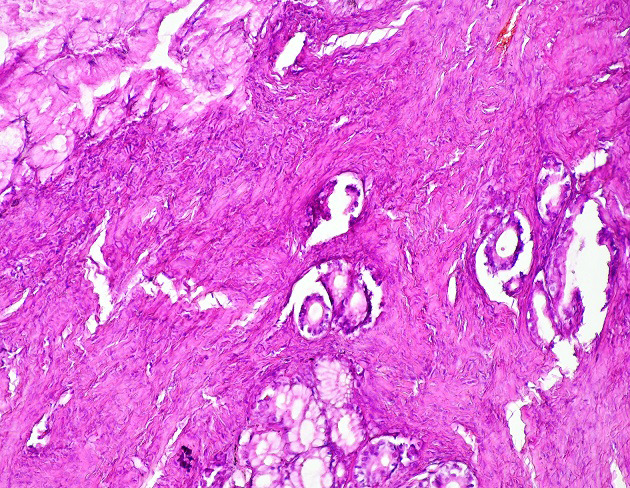
HEx250: mucinous adenocarcinoma of the ovary, mucinous glands in a fibrous stroma

A 57-year-old female patient underwent total thyroidectomy. Histopathology revealed a follicular thyroid carcinoma (pT1bNxMx). Four weeks later she received, with thyroid hormone withdrawal, 3.7 GBq of 131I as a treatment. At this time, the serum thyroglobulin level was 2.7 ng/mL, TSH was 73 µIU/ml and antithyroglobulin antibody level was less than 20 IU/mL. Five days after the treatment, WBS (Figure 1) showed mild uptake in the neck, representing thyroid remnants, and a large and a high heterogeneous radioiodine accumulation in the median lower abdomen and pelvis confirmed by Single Photon Emission Computed Tomography (SPECT) (Figure 2). An ultrasound showed a right adnexal mass measuring 126 mm with a double tissue and cystic components with heterogeneous vascularization on color Doppler (Figure 3). The patient underwent a hysterectomy with bilateral oophorectomy. Pathological examination found a mucinous adenocarcinoma of the right ovary (Figure 4).

## Discussion

Ovarian radioiodine uptake at post-therapy WBS may occur in benign or malignant conditions [2-4]. Pathology examination is the only way to differentiate between the two conditions. The benign pathological diagnosis can be a benign thyroid tissue (*struma ovarii*) [2], a benign mucinous ovarian cystadenoma [5, 6] and an ovarian endometriosis cyst [2]. The malignant conditions were metastasis of thyroid cancer cells to ovary and thyroid cancer originating from embryonic thyroid tissue in the ovary, which may also result in focal ovarian uptake [2]. To our knowledge, this is the first case of false-positive radioiodine uptake in an ovarian mucinous adenocarcinoma. The suggested mechanisms of radioiodine uptake in the ovarian cystadenoma include increased vascularity and capillary permeability [7].

## Conclusion

It is important to recognize the physiological and pathological aetiologies (unrelated to thyroid) that demonstrate 131 I uptake and may lead to false positif 131 I scan in patients of DTC.

## References

[1] Haugen BR, Alexander EK, Bible KC, Doherty MD, Mandel SJ, Nikiforov YR (2016). 2015 American Thyroid Association Management Guidelines for Adult Patients with Thyroid Nodules and Differentiated Thyroid Cancer: The American Thyroid Association Guidelines Task Force on Thyroid Nodules and Differentiated Thyroid Cancer. Thyroid.

[2] Jong-Ryool Oh and Byeong-Cheol Ahn (2012). False-positive uptake on radioiodine whole-body scintigraphy: physiologic and pathologic variants unrelated to thyroid cancer. Am J Nucl Med Mol Imaging.

[3] Vincenzo Triggiani, Vito Angelo Giagulli, Michele Iovino, Giovanni De Pergola, Brunella Licchelli, Antonio Varraso, Franca Dicembrino, Guido Valle, Edoardo Guastamacchia (2016). False positive diagnosis on 131iodine whole-body scintigraphy of differentiated thyroid cancers. Endocrine.

[4] Leckzinscka Buton, Olivier Morel, Patricia Gault, Frédéric Illouz, Patrice Rodien, Vincent Rohmer (2013). False-positive Iodine-131 whole-body scan findings in patients with differentiated thyroid carcinoma: Report of 11 cases and review of the literature. Ann Endocrinol.

[5] Zhong-Ling Qiu, Yan-Hong Xu, Hong-Jun Song, Quan-Yong Luo (2010). Unusual 131 uptake in a benign mucinous cystadenoma of the ovary in a patient with papillary thyroid cancer. Clin Nucl Med.

[6] Fatema Alzahraa Almohamad, Tareq Ahmad, Basel Ahmad, Khalid Hussain, Lama Hadid, Majdi Zein, Mohamad Ahmad (2018). False-positive radioiodine accumulation in a huge pelvic mass after thyroidectomy for papillary carcinoma, a case report from Syria. J Surg Case Rep.

[7] Brahm Shapiro, Vittoria Rufini, Ayman Jarwan, Onelio Geatti, Kimberlee J. Kearfott, Lorraine M. Fig, lan D. Kirkwood, Milton D. Gross (2000). Artifacts, anatomical and physiological variants, and unrelated diseases that might cause false-positive whole-body 131-I scans in patients with thyroid cancer. Semin Nucl Med.

